# Personal and Environmental Characteristics Associated with Choice of Active Transport Modes versus Car Use for Different Trip Purposes of Trips up to 7.5 Kilometers in The Netherlands

**DOI:** 10.1371/journal.pone.0073105

**Published:** 2013-09-05

**Authors:** Eline Scheepers, Wanda Wendel-Vos, Elise van Kempen, Luc Int Panis, Jolanda Maas, Henk Stipdonk, Menno Moerman, Frank den Hertog, Brigit Staatsen, Pieter van Wesemael, Jantine Schuit

**Affiliations:** 1 VU University Amsterdam, Department of Health Sciences, Amsterdam, The Netherlands; 2 National Institute for Public Health and the Environment, Centre for Nutrition, Prevention and Health Services, Bilthoven, The Netherlands; 3 National Institute for Public Health and the Environment, Centre for Sustainability, Environment and Health, Bilthoven, The Netherlands; 4 Transportation Research Institute (IMOB), Hasselt University, Diepenbeek, Belgium; 5 Flemish Institute for Technological Research (VITO), Mol, Belgium; 6 VU University Medical Centre, EMGO Institute, Amsterdam, The Netherlands; 7 SWOV Institute for Road Safety Research, Leidschendam, The Netherlands; 8 Inbo Amsterdam, Amsterdam, The Netherlands; 9 National Institute for Public Health and the Environment, Centre of Health and Society, Bilthoven, The Netherlands; 10 Technical University Eindhoven, Department Architectural Design & Engineering, Eindhoven, The Netherlands; Fundación para la Prevención y el Control de las Enfermedades Crónicas No Transmisibles en América Latina (FunPRECAL), Argentina

## Abstract

**Introduction:**

This explorative study examines personal and neighbourhood characteristics associated with short-distance trips made by car, bicycle or walking in order to identify target groups for future interventions.

**Methods:**

Data were derived from ‘Mobility Research Netherlands (2004–2009; MON)’, a dataset including information regarding trips made by household members (n = ±53,000 respondents annually). Using postal codes of household addresses, MON data were enriched with data on neighbourhood typologies. Multilevel logistic modelling was used to calculate odds ratio (OR) of active transport versus car use associated with four different trip purposes (shopping (reference), commuting, taking or bringing persons or sports). A total of 277,292 short distance trips made by 102,885 persons were included in analyses.

**Results:**

Compared to women shopping, women less often take active transport to sports clubs (OR = 0.88) and men less often take active transport for shopping (OR = 0.92), or for bringing or taking persons (OR = 0.76). Those aged 25–34 years (OR = 0.83) and 35–44 years (OR = 0.96) were more likely to use active transport for taking or bringing persons than persons belonging to the other age groups (relative to trips made for shopping by those 65 years or over). A higher use of active transport modes by persons with an university or college degree was found and particularly persons living in urban-centre neighbourhoods were likely to use active transport modes.

**Conclusion:**

In developing policies promoting a mode shift special attention should be given to the following groups: a) men making short distance trips for taking or bringing persons, b) women making short distance trips to sport facilities, c) persons belonging to the age groups of 25–44 years of age, d) Persons with a primary school or lower general secondary education degree and persons with a high school or secondary school degree and e) persons living in rural or urban-green neighbourhoods.

## Introduction

Physical inactivity is one of the main health problems in Western countries. According to the WHO, healthy adults aged 18 to 64 years should engage in at least 150 minutes of moderate-intensity aerobic physical activity throughout the week or engage in at least 75 minutes of vigorous-intensity aerobic physical activity throughout the week or an equivalent combination of moderate and vigorous activity [1]. Globally, almost 31% of the adults aged 15 years and older fail to do so [2]. Physical inactivity is estimated to cause around 21–25% of the burden of disease from breast and colon cancer, 27% of the burden of disease from diabetes and about 30% of ischeamic heart disease burden [3]. Therefore, interventions effectively stimulating physical activity are of great importance.

One way to stimulate physical activity is by affecting travelers’ choices regarding transport modes in favor of active modes (walking or cycling for the purpose of going somewhere). Several studies have shown that active transport has a protective effect on cardiovascular outcomes [4] and is inversely associated with Body Mass Index (BMI), obesity, triglyceride levels and insulin levels [5]. The Toronto Charter for Physical activity states that transport policies and systems that prioritize active transport are one of the best investments for stimulating physical activity since active transport is the most practical and sustainable manner to increase physical activity on a daily basis [6]. Increasing physical activity levels by stimulating active transport modes is expected to, and to a large extent has proven to be effective because active transport is linked to all domains of physical activity (occupational, domestic, transportation and leisure time physical activity) [7]–[9] and can be built into every day living [6], [10]. For example, Engbers *et al.* [7], who investigated the cycling behaviour of 799 Dutch participants with a mean age of 41 years, showed that 26% met the physical activity guideline simply by cycling to work.

Furthermore, stimulation of active transport use and related to this a decrease in car use not only influences the level of physical activity, but also has beneficial health effects due to reduced air pollution emissions [11], green house emissions [12] and noise levels [13]. The effect on road safety is claimed to be an improvement by Jacobsen [14], but other authors showed that this effect depends on age and gender [15]. Because of this diversity in (beneficial) effects on health and environmental quality, collaboration between the health, transport, spatial planning and environmental sector will be beneficial in developing cost-efficient interventions stimulating active transport.

Recently, stimulating active transport by enhancing the substitution of short distance car trips with walking or cycling trips has become a popular policy strategy. Several attempts have been made to determine the health impact of such a modal shift [9], [11]–[13], [16]. In a previous study [13], we attempted to determine the health effects of this modal shift, defining short distances as distances up to 7.5 km and assuming 10% of the adult population effectively substitutes their short distance car trips with cycling. Main results were a decrease in disease burden, expressed in Disability-Adjusted Life Years (DALYs), related to physical (in)activity at a maximum of 1.3% after one year. Small health benefits were found resulting from a reduction in road traffic noise levels and traffic-related air pollution and a possible health deficit could come from a higher risk of accidents. The main drawback of this study was that much of the information needed for this assessment was unavailable or unknown, resulting in a relatively high number of assumptions. This raised the question whether or not the estimated health effects were a correct representation of the outcome of this intervention in practice. One of the topics in need of elucidation was which groups within the adult population were indeed making short-distance trips by car and in which types of environments short-distance trips were more likely to be made by active transport modes [13].

Behaviour, such as cycling and walking, is affected by several personal and environmental factors. The intention to behave in a certain way is affected by attitudes, social norms and self-efficacy, which in turn predicts the behaviour [17]. These determinants of behaviour are influenced by personal characteristics (for example age, gender and educational level). The moment when and the reason why the intention is turned into actual behaviour is influenced by barriers and skills. An important set of barriers lies within the environment or more specifically, in the interaction between individuals and their environment (for example neighbourhood typology) [13].

The Netherlands are characterized by a very high share of active transport trips relative to other European countries. Nevertheless, there are still health benefits to be gained by stimulating even more active transport use [13]. With a raising awareness of the importance of stimulating active transport use around the world, insight in the factors positively influencing active transport use is needed. It may be expected that for different population groups different trip purposes are related to the use of active transport modes and that certain living environments are more facilitating than other regarding active transport modes. In the present study we examined the association between trip purposes and the choice of active (cycling and walking) versus passive (car) transport modes for short distance trips (up to 7.5 km; for explanation see methods) and explored the influence of personal and neighbourhood characteristics on this association. Policies promoting a shift from passive towards active transport modes are potentially more effective when they focus on frequent car users. Aim of the present study was to identify populations that may benefit most from interventions to promote active transport.

## Methods

### Data Sources

The main data source for this study was Mobility Research Netherlands (MON). The MON is an annual household survey of mobility behavior among inhabitants of the Netherlands commissioned by the Dutch Ministry of Transport, Public Works, and Water Management. Each year, a random sample of households in the Netherlands receive a letter explaining the aim of the study as well as aspects concerning data use and safety, a household questionnaire, travel diaries for each household member, and a return envelope. Based on this letter, participants can decide if they are willing to participate. In the travel diary, participants are asked to report all trips they made during one designated day. For each trip, additional information is gathered concerning mode of transport, trip purpose, start and end times of the trips, geographical location, as well as individual and household socio-demographics. Data is available for researchers via http://easy.dans.knaw.nl.

For the present study, data collected in the years between 2004 and 2009 was used. Travel diaries were completed for every day of the year with an equal distribution of households over all days of the year. A total of 317,258 persons participated in this study, together making more than a million trips. If participants were not able to fill in the paper versions of the questionnaire and travel diary, the possibility for an interview by telephone was provided. A total of 5,505 persons (6% of all persons included) used this option, with no large difference in response mode over years. More information about Mobility Research Netherlands can be found elsewhere [18], [19].

The MON dataset was merged with a dataset from ABF Research (2009) by using the four-digit postal codes of household addresses, complementing this dataset with information concerning neighbourhood typologies [20]. In the Netherlands there are 4000 four-digit postal codes, representing on average 1,772 households each. In urban areas this postal code represents only one neighbourhood, whereas in rural areas this postal code can represent a whole village.

### Selection of the Study Population

**Figure 1:**
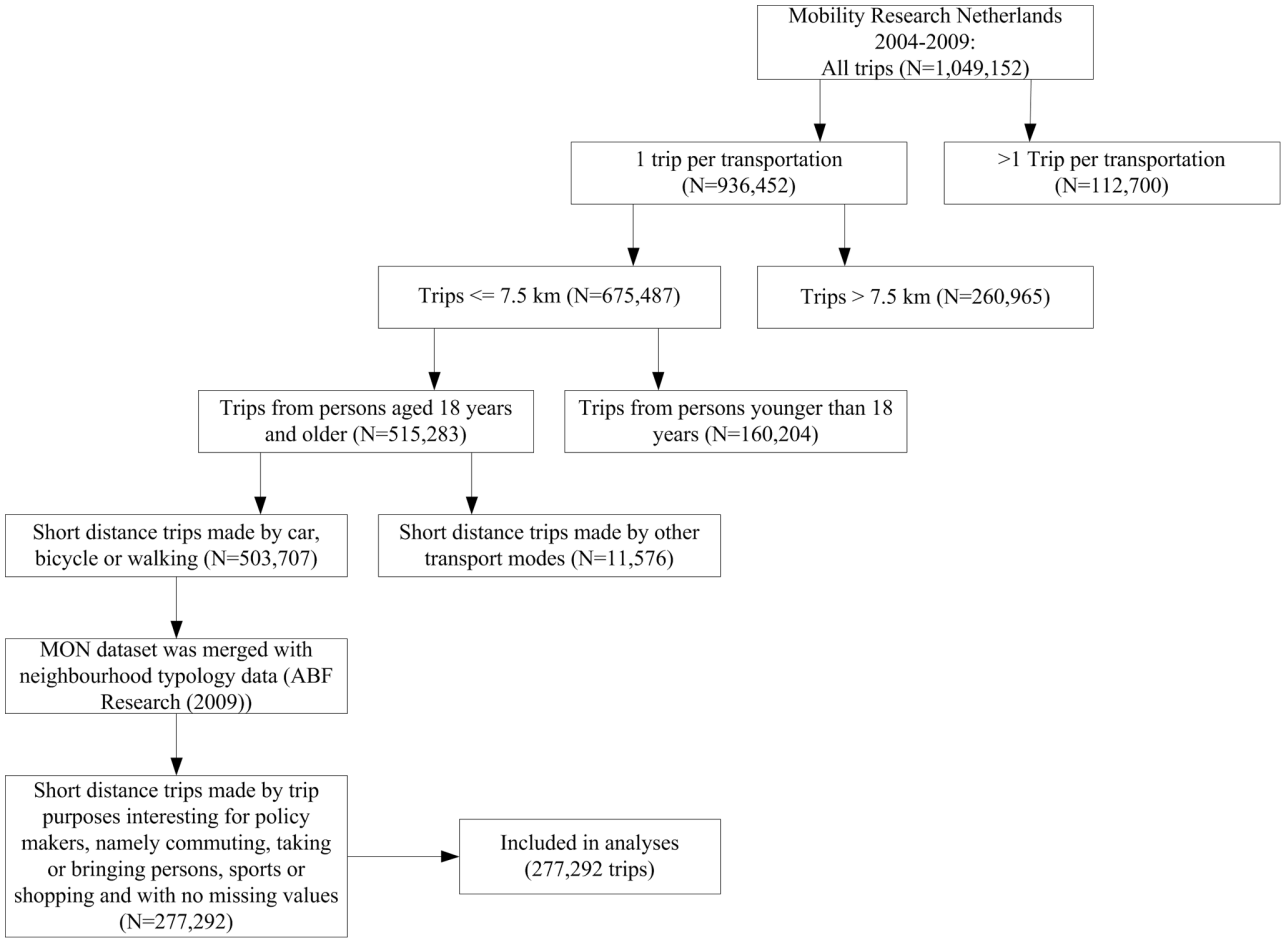
Flowchart trips included in analyses. No missing values on (1) trip purpose, (2) ownership of a car or bicycle, (3) age, (4) gender, (5) education level, (6) neighbourhood typology, (7) year and month of measurement or (8) postal code.

Figure 1 contains information about the selection of our study population from the merged dataset that was described above. In a number of cases, the trips in the dataset were part of a sequence of trips building up to a larger transportation. In order to keep our analyses and the interpretation of our results as straight forward as possible, only ‘single trips’ were included in the analyses (N = 936,452). Another restriction was made by selecting only trips up to a maximum distance of 7.5 km (short distance trips; N = 675,487). This distance was chosen since it represents 30 minutes cycling at an average cycling speed and thus would be sufficient to meet a daily part of the earlier mentioned physical activity guideline when a car trip of this maximal distance would be substituted by a cycling trip. Subjects younger than 18 years old were excluded from this study, because in the Netherlands below this age people do not have a driver’s license and therefore are not part of the target population for interventions stimulating a modal shift from car use towards active transport. This left us with 515,283 short distance trips. For the present study only short distance trips made by car, bicycle or foot were relevant (N = 503,707 trips).

### Trip Purposes

The present study focused on trip purposes interesting for policy makers and developers of intervention measures. The selected trip purposes are of interest because they imply a clear set of stakeholders and partners involved in case an intervention or policy measure in this domain is considered. The selected purposes were:

1. Shopping.
2. Commuting: trips made with the purpose of going to work as well as going to volunteer work.
3. Taking or bringing persons: trips made with the purpose of taking or bringing persons to regularly visited places, for example bringing children to school or sports.
4. Sports.

### Personal Characteristics

Personal characteristics included in this study were gender, age, educational level, car ownership (yes/no) and bicycle ownership (yes/no). Age was divided into six 10-year age categories: 18–24 years, 25–34 years, 35–44 years, 45–54 years, 55–64 years, and 65 years and older. Educational level was divided into three categories:

1. Persons with a primary school or lower general secondary education degree
2. Persons with a high school or secondary school degree
3. Persons with an university or college degree.

### Environmental Characteristics

In the present study the variable ‘neighbourhood typology’ was used. This variable poses a combination of various environmental characteristics. The data source (ABF Research) provided five different typologies based on density, accessibility/connectivity, land use mix and quality of buildings:

1. Urban – centre: city centres as well as some neighbourhoods just outside the centre.
2. Urban – outside centre: neighbourhoods with a larger distance from the city centre but with a higher density than the neighbourhoods classified as ‘urban-green’. Most of the time the density of these neighbourhoods is also higher than in the urban-centre neighbourhoods.
3. Urban – green: neighbourhoods classified as urban-green are predominantly residential areas. Density is lower than the mean density based on housing supply. New housing estate neighbourhoods are also classified as urban-green.
4. Village-centre: Neighbourhoods with a higher density or more facilities than rural neighbourhoods.
5. Rural: Neighbourhoods with a low density or relatively few facilities.

### Statistical Analyses

From the 503,707 short distance trips included in the dataset, a total of 226,415 trips were excluded from further analyses because they were not made with the purposes of shopping, commuting, taking or bringing persons, or sports or had missing values on the use of active transport modes or the following important covariates: trip purpose, ownership of a car or bicycle, age, gender, educational level, neighbourhood typology, year and month of measurement or postal code. Therefore, statistical analyses were based on a dataset with complete cases of 277,292 short distance trips (Figure 1).

To gain insight into how choice of transport mode (car, cycling, walking) was distributed over the various trip purposes these variables were cross-tabulated. The average trip distance and average travel time for short distance trips were calculated separately for using the car, cycling and walking. In addition, the proportion of people using both active and passive transport modes on the same day was calculated. To further characterize the study population, percentages of men and women within age-categories, categories of educational level and neighbourhood typologies were calculated as well as percentages of men and women owning a car, a bike and a driver’s license.

To investigate the association between trip purpose and choice of transport mode, multilevel logistic modeling (GLIMMIX procedure) was used to model the odds of using active transport (cycling and walking) versus passive transport (car) for the selected trip purposes. Multilevel modeling takes into account the hierarchical structure of the data (trips grouped within persons) and enables effects at both the level of person and trip to be included in the same model. Shopping (the main trip purpose) was used as reference category (ref). Adjustments were made for car ownership (yes/no (ref)), bicycle ownership (yes/no (ref)), season (winter, spring, summer, autumn (ref)), 10-year age categories (18–24 years, 25–34 years, 35–44 years, 45–54 years, 55–64 years and >64 years (ref)), gender (male/female (ref)), educational level (primary school or lower general secondary education degree, high school or secondary school degree, university or college degree (ref)) and neighbourhood typology (urban-centre (ref), urban-outside centre, urban-green, village-centre and rural), leading up to the following base model:
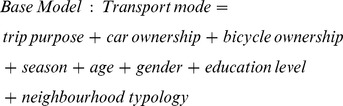



Coefficients (ß) and standard errors (SE) were estimated under residual pseudolikelihood estimations. From the resulting coefficients and standard errors, odds ratios (OR) and their 95% confidence intervals (CI) were calculated. The presented OR’s may be interpreted as the likelihood of an average person in our dataset choosing active transport over using the car for several trip purposes for trips up to 7.5 kilometers compared to shopping.

To investigate the influence of personal and neighbourhood characteristics on the association between trip purpose and choice of transport mode, we studied the interaction (F-test) between (1) trip purpose and gender, (2) trip purpose and age, (3) trip purpose and educational level, and (4) trip purpose and neighbourhood typology by using the following models: 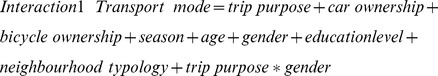


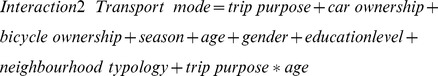


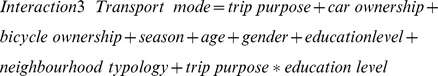


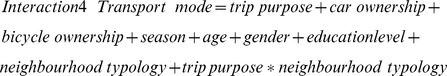



If significant, the odds ratios based on the interaction models were tabulated. Reference categories were shopping combined with either being female, being 65 years or older, having an university or college degree or living in an urban-centre neighbourhood. Data was analyzed using SAS version 9.3.

## Results

### Descriptive Statistics

A total of 277,292 short distance trips made by 102,885 persons were included in the analysis. Table 1 shows the distribution of these trips over transport modes in general and stratified by trip purpose. Main trip purposes were shopping (51%) and commuting (25%). Irrespective of trip purpose, approximately 44% of short distance trips were made by car. Bicycle use varied from 35% for taking or bringing persons and shopping to 47% for commuting purposes. For all trip purposes, walking was least prevalent with a range of 9% (commuting) to 21% (shopping). Short car trips had a larger average (average ± standard deviation) travel distance (3.3±1.9 km) than trips made by bicycle (2.1±1.6 km) or walking (0.8±0.8 km; not in Table). The average travel times for the trips made by car, cycling and walking trips were 9.8±6.7 minutes, 10.4±8.0 minutes and 9.8±9.1 minutes respectively. 20,457 persons (20%) used both active and passive transport modes on the same day (not in Table).

**Table 1:** Percentage of trips made by transport modes stratified by trip purpose.

	CommutingN = 69,916 (25%)	Taking or bringingpersons N = 43,989 (16%)	SportN = 20,756 (8%)	ShoppingN = 142,631 (51%)	TotalN = 277,292
**Transport mode (%)**					
Car	44	44	44	44	44
Bicycle	47	35	42	35	39
Walking	9	21	14	21	17

Table 2 shows the characteristics of the study population, making short distance trips by car, bicycle and/or foot included in our analyses. The study population consisted of more women (57%) than men (43%), with a comparable distribution over age categories and neighbourhood typologies. A primary school or lower general secondary education degree was more prevalent among women (42.8%) than among men (36.4%). Men, more often than women owned a car and a driver’s license (Table 2).

**Table 2:** Characteristics of the persons making short distance trips (N = 102,885).

		Men (N = 44,183)	Women (N = 58,702)
**Age (%)**	*18–24 years*	6.45	5.30
	*25–34 years*	13.06	14.92
	*35–44 years*	21.13	23.72
	*45–54 years*	20.58	21.56
	*55–64 years*	19.75	18.04
	*>64 years*	19.03	16.46
**Education level (%)**	*Primary school or lower general secondary degree*	36.37	42.76
	*High school or secondary school degree*	34.22	34.38
	*University or college degree*	29.41	22.86
**Car owners (%)**		75.85	51.16
**Bike owners (%)**		93.19	93.10
**Driver’s license owners (%)**		92.42	82.17
**Neighbourhood typology (%)**	*Rural*	10.55	10.51
	*Village-centre*	35.41	36.15
	*Urban-green*	13.77	13.43
	*Urban-outside centre*	32.98	32.94
	*Urban-centre*	7.29	6.97
**Season (%)**	*Winter*	24.17	23.68
	*Spring*	23.57	23.38
	*Summer*	23.43	23.82
	*Autumn*	28.83	29.12

### Association between Trip Purpose and use of Transport Modes

All confounders in itself were associated with choice of transport mode. Taking shopping as reference, commuting, taking or bringing persons and sports were purposes for which it was more likely that active transport modes were chosen over car use (Table 3).

**Table 3:** The association between trip purpose and choice of active transport mode (N = 277,292).

	Base model
	OR (95% CI)
**Trip purposes**	
*Commuting*	1.25 (1.23–1.28)
*Taking or bringing persons*	1.29 (1.27–1.32)
*Sport*	1.06 (1.04–1.09)
*Shopping*	1.00
**Car owners**	0.23 (0.22–0.23)
**Bicycle owners**	2.23 (2.11–2.34)
**Season**	
*Winter*	0.84 (0.81–0.87)
*Spring*	0.95 (0.92–0.98)
*Summer*	1.04 (1.01–1.08)
*Autumn*	1.00
**Age (years)**	
*18–24*	0.60 (0.56–0.64)
*25–34*	0.55 (0.53–0.58)
*35–44*	0.61 (0.59–0.64)
*45–54*	0.65 (0.62–0.68)
*55–64*	0.75 (0.71–0.78)
*>64*	1.00
**Men**	0.92 (0.90–0.95)
**Education level**	
*Primary school or lower general secondary degree*	0.88 (0.85–0.91)
*High school or secondary school degree*	0.84 (0.81–0.87)
*University or college degree*	1.00
**Neighbourhood typology**	
*Rural*	0.37 (0.35–0.40)
*Village-centre*	0.58 (0.55–0.61)
*Urban-green*	0.52 (0.49–0.55)
*Urban-outside centre*	0.65 (0.61–0.68)
*Urban-centre*	1.00

Adjusted for demographic and environmental factors.

Abbrevations; OR = Odds Ratio indicating the odds to use active transport modes for a short trip; 95% CI = 95% confidence interval; significance was tested at α = 0.05.

### Interaction between Trip Purpose and Personal Characteristics

**Figure 2:**
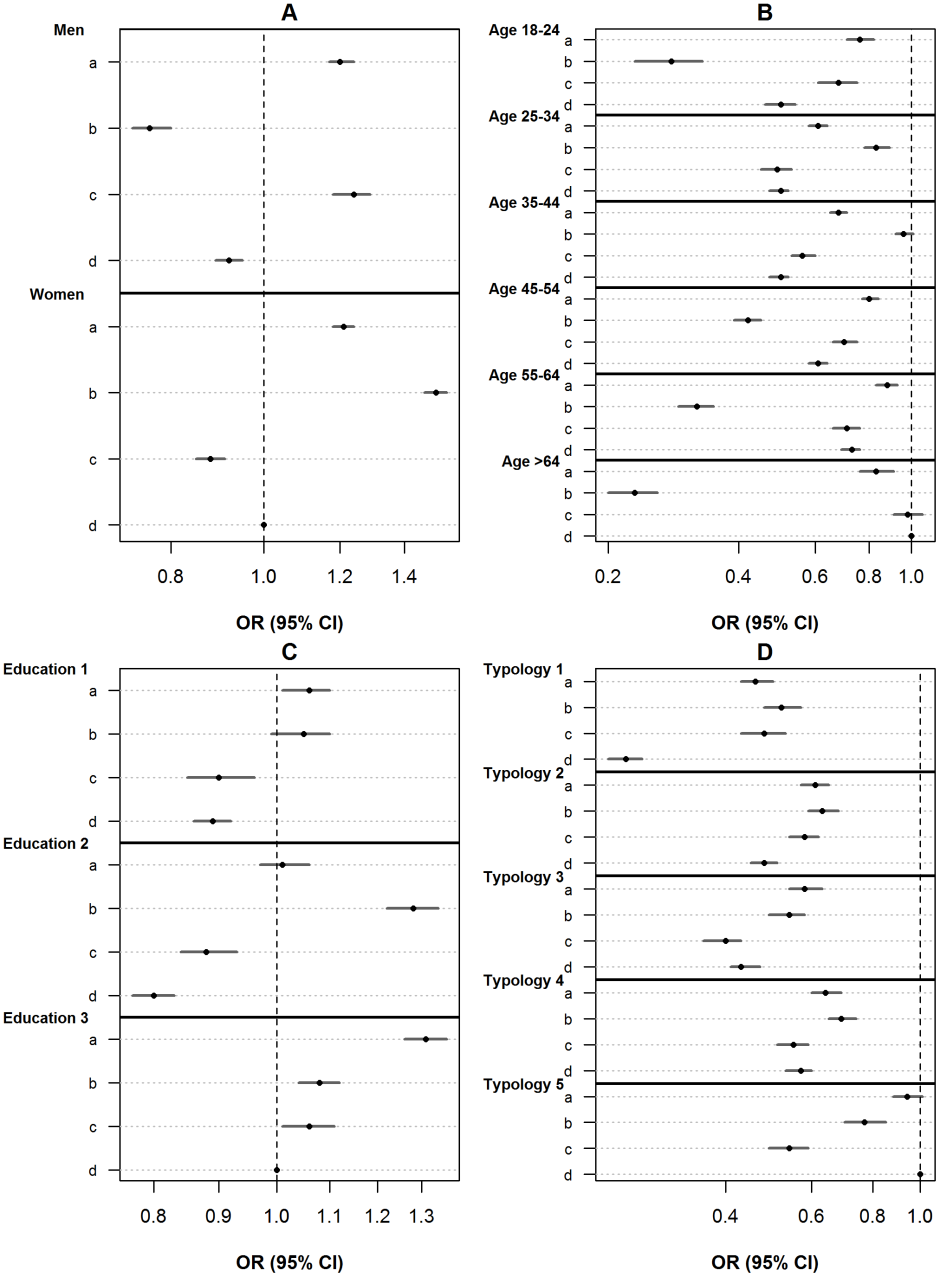
Association between trip purpose and choice of active transport mode for gender (A), age (B), education level (C) & neighbourhood typology (D). Trip purposes were presented by a – d, with a = commuting, b = taking or bringing purposes, c = sports, d = shopping. Figure A: adjusted for car ownership, bicycle ownership, season, education level, age and neighbourhood typology, with shopping trips made by women as reference category. Figure B: adjusted for car ownership, bicycle ownership, season, gender, education level and neighbourhood typology, with shopping trips made by persons being 65 years or older. Figure C: education 1 = persons with a primary school or lower general secondary education degree; education 2 = persons with a high school or secondary school degree; education 3 = persons with an university or college degree; Adjusted for car ownership, bicycle ownership, season, gender, education level, age and neighbourhood typology, with shopping trips made by persons having an university or college degree as reference category. Figure D: typology 1 = rural; typology 2 = village-centre; typology 3 = urban-green; typology 4 = urban-outside centre; typology 5 = urban-centre; Adjusted for car ownership, bicycle ownership, season, gender, age, education level, with shopping trips made by persons living in an urban-centre neighbourhood as reference category.

Significant interactions between trip purpose and personal characteristics were found for gender (F = 334.15, Num DF = 3, Den DF = 20689, p = <0.0001), age (F = 199.77, Num DF = 15, Den DF = 20677, p = <0.0001) and educational level (F = 51.85, Num DF = 6, Den DF = 20686, p = <0.0001). Figure 2A shows that, in comparison with women shopping, men shopping (OR = 0.92, 95% CI: 0.89–0.95), men taking or bringing persons (OR = 0.76, 95% CI: 0.73–0.80) and women making a short distance trip for the purpose of sports (OR = 0.88, 95% CI: 0.85–0.91) were less likely to make use of active transport, but used the car. The most profound differences between men and women were found for taking or bringing persons (men: OR = 0.76, 95% CI: 0.73–0.80); women: OR = 1.51, 95% CI: 1.47–1.55) and sports (men: OR = 1.24, 95% CI: 1.18–1.29; women: OR = 0.88, 95% CI: 0.85–0.91).

Comparison with shopping trips made by persons aged 65 years and older, showed all other combinations of trip purpose and 10-year age category to be more likely made by car than by active transport, with the exception of trips made for the purpose of taking or bringing persons by people aged between 35 and 44 years and trips made with the purpose of sports by people aged 65 years or over for which the OR did not reach statistical significance (Figure 2B). For the purpose of shopping, commuting and sports, OR’s for active versus passive transport (relative to short distance trips made by those 65 years or over for the purpose of shopping) generally increased with increasing age. Exceptions were trips made by those aged 18–24 years for the purposes of commuting and sports for which OR’s were higher compared to the adjacent age category. The association between the purpose of taking of bringing persons and choice of transport mode shows a deviant pattern across age categories. OR’s of active versus passive transport increase from the age category of 18–24 years (OR = 0.28, 95% CI: 0.23–0.33) up to the age category of 35–44 years (OR = 0.96, 95% CI: 0.92–1.01) and after that show a rapid decrease up to the highest age category (>64 years: OR = 0.23, 95% CI: 0.20–0.26).

Figure 2C, shows that for shopping, commuting and sport purposes the same pattern can be seen, with persons with a university or college degree using active transport most and a lowest use of active transport by persons with a high school or secondary school degree. However, only for shopping trips a significant difference was found between persons with a primary school or lower general secondary education degree (OR = 0.89, 95% CI: 0.86–0.92) and persons with a high school or secondary school degree (OR = 0.80, 95% CI: 0.77–0.83) persons. The higher use of active transport modes by persons with a university or college degree was most pronounced by trips made with a commuting purpose (OR = 1.31, 95% CI: 1.26–1.36). Trips made with the purpose of taking or bringing persons were more likely to be made by active transport by persons with a high school or secondary school degree (OR = 1.28, 95% CI: 1.22–1.34) compared to persons with a primary school or lower general secondary education degree (OR = 1.05, 95% CI: 0.99–1.10) and persons with a university or college degree (OR = 1.08, 95% CI: 1.04–1.12). Combinations of trip purpose and educational level specifically associated with less use of active transport (OR <1) were short distance trips made for the purpose of shopping and sports by those with a primary school or lower secondary school degree and those with at a high school or secondary school degree. Combinations specifically associated with higher use of active transport (OR >1) were short distance trips made for the purpose of commuting by those with an university or college degree and short distance trips made for the purpose of taking or bringing persons by those with a high school or secondary school degree.

### Interaction between Trip Purpose and Neighbourhood Typology

A significant interaction was found between trip purpose and neighbourhood typology (F = 59.68, Num DF = 12, Den DF = 20680, p<0.0001). Interaction model 4 showed that, compared to shopping trips made by persons living in urban-centre neighbourhoods, all other combinations of trip purpose and neighbourhood typology were less likely to represent active transport trips (Figure 2D). Specifically trips made for the purpose of sports, showed a different distribution across neighbourhood typologies with low odds ratios in urban-outside centre and urban centre neighbourhoods relative to village-centre neighbourhoods (not significant). For all trip purposes lower odds ratios were found for persons living in urban-green neighbourhoods compared to persons living in village-centre or urban-outside centre neighbourhoods (Figure 2D).

## Discussion

Our findings indicate that trip purpose is an important predictor for the use of active transport versus passive transport modes. Taking shopping as a reference, all short distance trips made with the purpose of commuting, taking or bringing persons and sports were more likely to be made by active transport modes than by car. Furthermore, this study showed that, given a certain trip purpose, gender, education level, age and neighbourhood typology have a further predictive value. Combinations of trip purpose and categories of demographic variables showed that, relative to women shopping, taking the car was more likely than using active transport for men shopping or taking or bringing persons and women making short distance trips for the purpose of sports. Relative to those aged 65 years or over making short distance trips for the purpose of shopping, all other combinations of age and trip purpose were more likely to represent short distance trips made by car than by active transport. With respect to educational level it was shown that short distance trips made for the purpose of shopping and sports by persons with a primary school or lower secondary school degree and by persons with at a high school or secondary school degree were more likely to be made by car than by active transport. Combinations of trip purpose and neighbourhood typology showed that, in general, neighbourhood typologies representing a higher density and accessibility were associated with a higher likelihood of short distance trips made by active transport than by using the car.

### Gender Differences

It was shown that men use active transport modes more often to go to sport facilities than women. Further exploration of our dataset learned that women tend to make short distance trips for the purpose of sports between 6 pm and midnight (41% of these trips). Possibly, personal and/or social safety aspects are factors of interest in this association [21]. In line with this, a study of a travel survey performed in San Francisco, showed that nightfall deterred walking [22].

Another gender difference was seen for the trip purpose taking or bringing persons. Women were more likely to use active transport modes for this trip purpose. A possible explanation for this gender difference might be that men make these trips more often in combination with other trip purposes (combined trip purposes). However, no information about these combined trip purposes was available in the present study. Future research should focus more on these combined trips, since it would be expected other aspects will influence mode of transport choice for these trips.

In contrast to a study of Vandenbulcke *et al.* [23] who investigated travel behaviour at 589 municipalities in Belgium, in the present study no gender differences were found for commuting trips. This difference could be explained by the fact that in the Netherlands the cycling culture is more developed. In Belgium only 19% of the persons living within 5 km of their work used the bicycle as the main method of transport for commuting [23], in our study population 47% of the commuting trips were made by bicycle.

### Age Differences

An increase in active transport use was found with an increase in age for the trips made with the purpose of shopping, commuting and sports. The higher use of active transport modes by persons with an age of 65 years and older could be explained by stage of life. At this age, most persons are retired and thus have more time to engage in walking and/or cycling. Another factor possibly influencing this higher use of active transport modes is ownership of driver’s license. Almost 30% of the persons belonging to the group of 65 years and older did not own a driver’s license.

The increase in use of active transport modes for the purpose of taking or bringing persons from the age category of 18–24 years up to the age category of 35–44 years and related to this the high use of active transport modes by persons belonging to the age groups of 25–34 years and 35–44 years could be explained by household structure. At this age, persons often have young children. In the Netherlands, the mean age of giving birth to their first child is 29.4 years for the mother and 32.4 years for the father [24]. Most of the parents often bring their children to school (mean distance to primary school in 2009 was only 0.9 km). Related to this we observed that persons of this age made respectively 37% and 34% of the trips with the purpose of taking or bringing persons of distances shorter than 1 km (compared to ±20% for the other age groups).

The lower odds ratio values for trips made to go to sport facilities, by these two age groups, could also be explained by this household structure. Taking care for children is time consuming and less time is left to go to sport facilities. Related to this, it was found that 32% of the persons belonging to the age group of 25–34 years and 24% of the persons belonging to the age group of 35–44 years were making these trips between 8pm and midnight. Besides these time constraints, personal and/or social safety could also explain the lower active transport use between 8pm and midnight.

### Education Level Differences

No large differences in mode of transport were found between the three education groups. Persons with a university or college degree were found to be more likely to use active transport. In line with this Merom *et al*. [25] showed in a household survey conducted in Sydney, that persons belonging to the highest-income quintile were more likely to perform more than 10 minutes cycling per day. However, in this study of Merom *et al*. no differences between education levels were found for walking. In line with previous research [5], [26], this higher use of active transport by persons with a university or college degree was found to be most pronounced for trips made with the purpose of commuting. A possible explanation for this could be that wealthy persons pay more attention to their health and therefore cycle more [27].

Another explanation could be found in the symbolic and affective factors of the car. Steg [28] argued that the choice to use the car instead of active transport was not only made based on the instrumental value of the car, but also based on these symbolic and affective factors (e.g. status symbol, expression by means of their car). She found that especially lower income persons judged cars more favourably than did higher income groups [28]. This would explain the lower use of active transport modes by persons with a primary school or lower general secondary education degree.

Persons with a high school or secondary school degree were most likely to use active transport for taking or bringing persons compared to the other two education groups. However, in this study no information was available about the motivations for choosing a specific transport mode. Future research is needed to be able to better understand this higher use of active transport by persons with a high school or secondary school degree.

### Neighbourhood Typology

Neighbourhood typology was shown to be related to transport mode choice. Two fundamental aspects of land use mentioned to influence transport mode choice are proximity and connectivity [8]. We observed that persons living in urban-centre neighbourhoods were more likely to use active transport than persons living in neighbourhoods with other typologies for nearly all trip purposes (except sports). Similar results were found by a study investigating 343 Flemish people (Belgium) to examine psychosocial and environmental predictors of cycling for transportation [26]. A possible explanation for this could be that these neighbourhoods are characterized by a high density, proximity and connectivity and thus encourage more active transport (or discourage car use) [29]. Related to this it was seen that in rural neighbourhoods active transport was less often used, which might be explained by the fact that these neighbourhoods are characterized by a low density or relatively few facilities which results in less opportunities for short distance trips [21].

Persons living in urban-green neighbourhoods were less likely to use active transport than persons living in village-centre neighbourhoods. These urban-green neighbourhoods are often set out spaciously, which reduces facility density and increases the possibility of parking a car near one’s home [30]. Village-centre neighbourhoods are the centers of towns and are characterized with a higher density or more facilities than rural neighbourhoods. A higher proximity to facilities might explain the difference between these urban-green and village-centre neighbourhoods.

However, the observed association between neighbourhood typology and mode of transport might also be due to self-selection. With the cross-sectional data we cannot rule out that people might choose their living environment based on their travel behaviour [31]. Therefore, we cannot state that moving to an area with another neighbourhood typology will always result in a change in choice of transport mode.

### Strengths and Limitations

One of the strengths of this study is the large sample size. This enabled us to study detailed interactions. Another strength are the methods involving the travel diaries. Participants were asked to fill in the diary for a designated day. The advantage of setting a date in the future is that persons could actually take their diary with them on this day and fill in the diary after each trip made [32]. Also, it made sure that data was collected for all days of the year, which made correction for season possible.

With the used travel diaries, we gathered valuable information concerning transport modes used for different trip purposes. However, the travel diaries provided no information about why people chose a specific transport mode. Previous research has shown that cultural tradition plays a role in this choice of transport mode [21], as well as personal attitudes, motivation, and social support systems [33], [34]. Since these travel motives are a connection point for stimulating bicycle use, future research should focus on these personal attitudes, motivations, and social support systems. Both quantitative as well as qualitative research (for example focus groups) is needed to be able to elucidate on these personal attitudes and motivations.

Data from the MON was enriched with data from living environment characteristics based on the four-digit postal codes of the household addresses. However, not all trips that are involved in this study started at the household address as well as environmental characteristics of the destination may differ from the characteristics at the household address. Future research should also focus on the environmental factors related to the route chosen and trip destinations, since these might also influence mode of transport.

In the present study only a rough classification of neighbourhood typologies was used to determine if there were some environmental characteristics related to transport mode choice. This has given some idea of what environmental aspects should be taken into account when thinking about interventions and policy measures regarding a mode shift. However, in the end more detailed information concerning environmental characteristics for developing neighbourhoods supporting active transport is needed. For these purposes, probably small-scale studies will be more suitable, since they may more easily yield detailed information on environmental characteristics than large scale studies such as this one.

## Conclusions

Identification of target groups was the aim of this study. Based on our results we can conclude that policy makers should take into account the impact of gender, age, education level and neighbourhood typology when developing measures to stimulate a shift from passive to active transport. More specifically, special attention should be given to the groups using little active transport, namely a) men making short distance trips with the purpose of taking or bringing persons, b) women making short distance trips to sport facilities, c) persons belonging to the age groups of 25–44 years of age, d) Persons with a primary school or lower general secondary education degree and persons with a high school or secondary school degree and e) persons living in rural or urban-green neighbourhoods. However, more detailed information is needed about these specific target groups and their living environment to develop suitable interventions and policy measures for stimulating a mode shift.
